# Invasion Genetics of the Western Flower Thrips in China: Evidence for Genetic Bottleneck, Hybridization and Bridgehead Effect

**DOI:** 10.1371/journal.pone.0034567

**Published:** 2012-04-03

**Authors:** Xian-Ming Yang, Jing-Tao Sun, Xiao-Feng Xue, Jin-Bo Li, Xiao-Yue Hong

**Affiliations:** Department of Entomology, Nanjing Agricultural University, Nanjing, Jiangsu, China; Brigham Young University, United States of America

## Abstract

The western flower thrips, *Frankliniella occidentalis* (Pergande), is an invasive species and the most economically important pest within the insect order Thysanoptera. *F. occidentalis*, which is endemic to North America, was initially detected in Kunming in southwestern China in 2000 and since then it has rapidly invaded several other localities in China where it has greatly damaged greenhouse vegetables and ornamental crops. Controlling this invasive pest in China requires an understanding of its genetic makeup and migration patterns. Using the mitochondrial COI gene and 10 microsatellites, eight of which were newly isolated and are highly polymorphic, we investigated the genetic structure and the routes of range expansion of 14 *F. occidentalis* populations in China. Both the mitochondrial and microsatellite data revealed that the genetic diversity of *F. occidentalis* of the Chinese populations is lower than that in its native range. Two previously reported cryptic species (or ecotypes) were found in the study. The divergence in the mitochondrial COI of two Chinese cryptic species (or ecotypes) was about 3.3% but they cannot be distinguished by nuclear markers. Hybridization might produce such substantial mitochondrial-nuclear discordance. Furthermore, we found low genetic differentiation (global *F*_ST_ = 0.043, P<0.001) among all the populations and strong evidence for gene flow, especially from the three southwestern populations (Baoshan, Dali and Kunming) to the other Chinese populations. The directional gene flow was further supported by the higher genetic diversity of these three southwestern populations. Thus, quarantine and management of *F. occidentalis* should focus on preventing it from spreading from the putative source populations to other parts of China.

## Introduction

Among the different ecological, genetic, and evolutionary features that determine whether an invasion will succeed or fail, genetic characteristics have received increasing attention [1]–[3]. For instance, introduced populations often show lower genetic diversity than do native populations, likely as a result of the founder effect [4]–[7]. Furthermore, successful invaders may often pass through only short-lived bottlenecks that are followed by rapid population expansions [8]. Thus, successive loss of genetic diversity would occur due to a specific evolutionary scenario termed the bridgehead effect, which refers to widespread secondary invasions stemming from a particular primary invasive population [9], [10]. Alternatively, enhanced genetic diversity of invasive species could arise due to multiple independent introductions and subsequent admixture, hybridization and introgression, which in turn may facilitate adaption [11], [12]. Therefore, an understanding of the genetic characteristics of invasive species is essential for understanding their performance in invaded habitats and the rapid evolution of invasiveness [3]. Such information is also critical to predicting the future movements of invasive species, preventing their further introduction, and controlling and eradicating them [13], [14].

The western flower thrips, *Frankliniella occidentalis* (Pergande), is an invasive species and the most economically important pest within the insect order Thysanoptera, which includes more than 5500 described species [15], [16]. *F. occidentalis* causes enormous damage by directly feeding on greenhouse vegetable and ornamental crops and by transmitting plant-pathogenic tospoviruses [17]. *F. occidentalis* is endemic to North America in an area west of the Rocky Mountains from Mexico to Alaska [18]. Over this range, Brunner & Frey [19] identified two ecotypes corresponding to different climate regimes. They might represent two cryptic species, between which reproductive isolation occurred, as suggested by Rugman-Jones et al. [20]. Since the late 1970s, *F. occidentalis* has rapidly invaded most countries in the world and now exists on every continent but Antarctica [21].

In China, *F. occidentalis* was first discovered in flowers from Myanmar at the Kunming International Floral Festival of China in 2000 [22], but it wasn't reported as an invasive species until 2003 when it was discovered in a greenhouse in Beijing [23]. Since then, it has rapidly spread to several provinces, including Yunnan, Heilongjiang, Shandong, Liaoning, Guizhou where it not only causes severe economic losses but also threatens endemic invertebrates and associated ecosystems [24]–[26]. In order to control *F. occidentalis*, it is first necessary to know its genetic diversity, population structure and pathways of range expansion in China.

Genetic tools, such as mitochondrial DNA (mtDNA) and microsatellites, can reveal the origins of newly established populations, their genetic makeup and their routes of migration [27], [28]. However, genetic studies of *F. occidentalis* have been hampered by a lack of polymorphic molecular markers. Presently, only 6 polymorphic microsatellites are known [29].

In this study, we isolated new polymorphic microsatellites markers for *F. occidentalis* and used them and the mitochondrial COI gene to characterize the genetic structure of 14 introduced populations of *F. occidentalis* in China. The primary goals of this study were to (1) investigate the genetic diversity and differentiation of introduced populations across China and (2) identify the potential avenues of dispersal and determine whether their range expansion in China is the result of a bridgehead effect.

## Materials and Methods

### Sample collection and species identification

**Figure 1:**
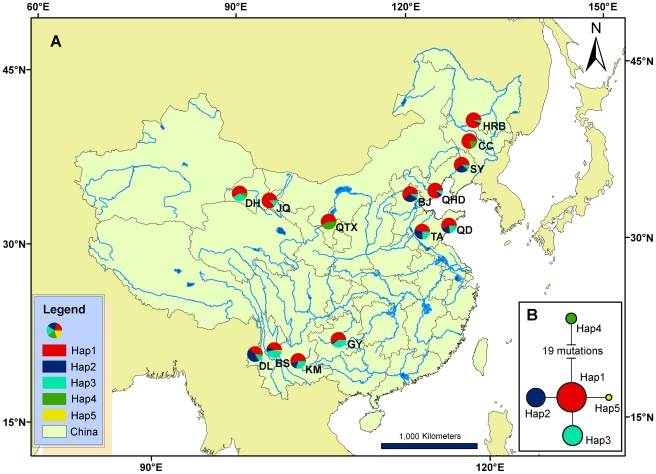
Sampling sites and haplotype frequencies in the examined populations of *F. occidentalis*. (A) Haplotype frequencies of COI in 14 populations in China. The abbreviations and coordinates of collection sites are shown in Table 1. (B) Haplotype network based on mitochondrial COI sequence. Frequency of haplotype is proportional to circle area. Each line between circles represents one mutational event.

In total, 506 *F. occidentalis* female adults were sampled representative of 14 sites in China during May 2009 to August 2010 (Table 1, Figure 1). The sampled localities cover a great part of the recent invasion areas ranging from HRB in the northeast to BS in the southwest of China. We caught the individuals either by beating the flowers, leaf over a white plastic tray or placing the flowers, leaf directly into jars with 95% ethanol separately. To limit the chance of sampling individuals that share the same parents, each thrips was collected from a single host plant separated by at least 1 m from the next sample. The collected individuals were examined and identified unambiguously using a dissecting microscope based on the characters such as the number, size and location of the major setae on the head, pronotum, forewing and abdominal tergite II, as well as colouration characteristics and comb of abdominal segment VIII [30], [31].

**Table 1:** Collection information for samples used in this study.

Code	Location	Nb samples	Coordinates	Sampling dates	Host
BJ	Beijing	48	39°57′34.52″N, 116°19′48.55″E	2 July 2010	*Phaseolus vulgaris.* L
DH	Dunhuang	24	40°08′22.25″N, 94°39′35.09″E	18–19 July 2009	*Tagetes erecta* L.
GY	Guiyang	30	26°39′46.08″N, 106°48′57.38″E	25 April 2009	*Petunia hybrida* Vilm; *Cucurbita moschata*; *Cucurbita pepo* L.
JQ	Jiuquan	35	39°46′42.82″N, 98°30′21.88″E	16–17 July 2009	*Tagetes erecta* L.
HRB	Harbin	44	45°44′30.54″N, 126°37′59.84″E	23 August 2009	*Tagetes erecta* L.; *Hosta ventricosa* (Salisb.) Stearn
QHD	Qinhuangdao	47	39°54′09.81″N, 119°32′18.92″E	27–28 August 2009	*Petunia hybrida* Vilm; *Canna indica* L.
CC	Changchun	10	43°53′08.63″N, 125°18′20.38″E	25 August 2009	*Hemerocallis fulva* (L.) L.;
SY	Shenyang	47	41°49′49.10″N, 123°34′09.65″E	26 August 2009	*Fuchsia hybrida* Voss; *Petunia hybrida* Vilm
QTX	Qingtongxia	7	37°54′24.57″N, 105°57′02.79″E	15–16 July 2009	*Althaea rosea*
QD	Qingdao	47	36°19′10.29″N, 120°23′32.18″E	1–2 June 2009	*Trifolium* L.; *Rosa chinensis*
TA	Taian	41	36°11′44.02″N, 117°07′12.76″E	30–31 May 2009	*Rosa chinensis*; *Tagetes erecta* L.
BS	Baoshan	48	25°10′24.55″N, 99°13′12.53″E	5 August 2009	*Solanum melongena* L.; *Brassica campestris* L.
DL	Dali	30	25°36′17.49″N, 100°14′49.75″E	7 August 2009	*Trifolium* L.; *Nicandra physalodes*; *Canna indica* L.; *Rosa chinensis*
KM	Kunming	48	24°42′43.91″N, 102°43′07.76″E	10–11 August 2009	*Dianthus caryophyllus*; *Trifolium* L.

Nb samples, number of samples.

No specific permits were required for the described field studies. (a) No specific permissions were required for these collections because the thrips are pests on common crops; (b) The location is not privately-owned in any way; (c) The field studies did not involve endangered or protected species.

### DNA extraction

Total genomic DNA was extracted by homogenizing a single female adult in a 50 µl mixture of STE buffer (100 mM NaCl, 10 mM Tris-HCl, 1 mM EDTA, pH 8.0) in a 1.5 ml Eppendorf tube. The mixture was incubated with 2 µl proteinase K (10 mg/ml) at 37°C for 30 min, followed by 5 min at 95°C. The samples were centrifuged briefly, and used immediately or stored at −20°C for the PCR reactions.

### Mitochondrial and nuclear DNA sequencing

All the individuals sampled were sequenced with primers C1-J-1751 and C1-N-2329 to yield a 571-bp fragment of the mitochondrial cytochrome c oxidase subunitIgene (COI) [32]. PCRs were performed on a Veriti machine (ABI Biosystems) in a 25 µl reaction volume containing 0.75 units of DreamTaq polymerase, 1× DreamTaq Buffer (including 2 mM MgCl_2_; Fermentas), 0.2 mM dNTPs (Takara), 1 µl of DNA (concentration not estimated) and 0.4 µM each of the oligonucleotide primers. The thermal profile used an initial denaturation step of 95°C for 3 min followed by 35 cycles of denaturing at 94°C for 30 s, annealing at 53°C for 30 s, and extension at 72°C for 45 s. A 7 min final extension at 72°C was added at the end of cycle to increase copy number. Negative controls were included in both DNA isolation and PCR reactions.

We also amplified a 456-bp fragment of the D2 domain of 28S (28SD2) nuclear ribosomal DNA from a sub-set of samples possessing the five different mtDNA haplotypes found in this study (see Results; at least 12 individuals for each haplotype (except Hap5) were sequenced) to compare our results to previous studies in its native range. The PCR was similar to the amplification of the COI mentioned above, except the annealing temperature at 50°C and the primer pairs, 28sF3633 (5′-TACCGTGAGGGAAAGTTGAAA-3′) and 28sR4076 (5′-AGACTCCTTGGTCCGTGTTT-3′) [33]. After verification via gel electrophoresis, the PCR templates were purified and then sequenced in both directions using the same primer pairs on an Applied Biosystems 3130 Genetic Analyzer.

### Microsatellite genotyping

Samples were genotyped at ten microsatellite loci developed for *F. occidentalis*. Eight new polymorphic microsatellites (WFT01-WFT08; Table S1 and Table 2) isolated from a genomic DNA library enriched for (TC)_6_(AC)_5_, (AC)_6_(TC)_5_ or (AC)_6_(AG)_5_ were used in this study, details of genomic library construction and microsatellite isolation was described by Lian et al. [34]. For the newly isolated microsatellite, each 15 µl genotyping PCR reaction volume containing 0.5 units of DreamTaq polymerase, 1× DreamTaq Buffer (including 2 mM MgCl_2_; Fermentas), 0.2 mM dNTPs (Takara), 1 µl of DNA (concentration not estimated), 0.3 µM forward primer, 0.3 µM fluorescent (FAM or HEX) labeled new ssr primer ((TC)_6_(AC)_5_, (AC)_6_(TC)_5_ or (AC)_6_(AG)_5_). The thermal profile used an initial denaturation step of 95°C for 3 min followed by 35 cycles of denaturing at 94°C for 30 s, annealing at 54°C for 30 s, and extension at 72°C for 45 s and a final 10 min extension at 72°C. Two additional microsatellites (FOCC75, FOCC125) were selected and PCR conditions were described previously by Brunner & Frey [29]. Products with different color and size range were combined and run in an Applied Biosystems 3130 Genetic Analyzer using LIZ-500 size standard. Data were collected and binned with GeneMapper v 4.0.

**Table 2:** Characteristics of eight new polymorphic microsatellite loci in *Frankliniella occidentalis*.

Locus	Repeat motif	Primer sequence (5′-3′)	Size range	Ta (°C)	*N*	*N*_A_	GenBank number
WFT01	(TC)6(AC)23	F: GAGGGAAATGGGAATCGTC R: TCTCTCTCTCTCTCACACAC	123–163	55	506	16	JN790701
WFT02	(TC)6 (AC)8	F: ATCGGTGACGAGTCACTTTG R: TCTCTCTCTCTCTCACACAC	131–143	55	506	7	JN790702
WFT03	(AC)6 (TC)7	F: ATTGCGCCGATTCCATGTC R: ACACACACACACACTCTCTC	86–96	55	506	6	JN790703
WFT04	(AC)6 (AG)21	F: GCGTGCCTCAAACCCTGTAC R: ACACACACACACACAGAGAG	105–162	55	502	18	JN790704
WFT05	(TC)6 (AC)5	F: TCGGCACTGTAATCGCATAT R: TCTCTCTCTCTCTCACACAC	118–146	55	505	11	JN790705
WFT06	(TC)6 (AC)21	F: CAAGCGTGTATCGCATAAG R: TCTCTCTCTCTCTCACACAC	138–201	55	497	23	JN790706
WFT07	(AC)6 (TC)9	F:GACCTTAGGGCAAATCTGAG R: ACACACACACACACTCTCTC	179–231	55	490	21	JN790707
WFT08	(AC)6 (TC)6	F: TTTGCTCGGCCTCGTTGTAG R: ACACACACACACACTCTCTC	129–137	55	505	5	JN790708

Ta, anneal temperature; *N*, Number of individuals with successful amplification; *N*_A_, total number of alleles.

### Data analysis

#### Sequence data

Sequences were assembled with CodonCode Aligner 3.6.1 (CodonCode, Dedham, MA, USA) and manually edited before creating consensus sequences. The resulting consensus sequences of all individuals were aligned using Clustal X 2.0.11 [35]. All population genetic parameters such as number of haplotypes (*N*_h_), nucleotide diversity (π) and haplotype diversity (*H*_d_) were calculated using the program DNASP v5 [36]. An analysis of molecular variation (AMOVA) implemented in Arlequin v3.01 [37] was used to test the hierarchic genetic structure of the populations. TCS v1.21 was used to generate a haplotype network using statistical parsimony [38].

#### Microsatellite data

The population genetic diversity indices such as total alleles per locus (*N*_A_), observed heterozygosity (*H*_O_), unbiased expected heterozygosity (*uH*_E_), mean number of alleles (*N*a), and number of private alleles (*N*_P_) were assessed using GenAlEx 6.41 [39]. The program Genepop 4.0.10 [40] was used to test for linkage disequilibrium between pairs of loci in each population (100 batches, 1000 iterations per batch) and for deviations from Hardy-Weinberg equilibrium (HWE) at each locus/population combination using Fisher's exact tests. Allelic richness (*A*_R_) was calculated with FSTAT 2.9.3.2 [41] using a rarefaction index (2N = 10) to account for different sample sizes. The program FSTAT was also used to calculate the gene diversity (*H*_S_). We used the one-side group comparisons in FSTAT with 1000 permutations to test for significant differences in allelic richness and gene diversity between the two groups inferred by population-pairwise *F*_ST_ analysis (see below).

Populations that have recently experienced a bottleneck should exhibit larger gene diversity than expected from the number of alleles at mutation–drift equilibrium since the allele numbers decrease faster than gene diversity in a recently bottlenecked population [42]. A possible significant heterozygosity excess (signature of bottleneck) was detected using a Wilcoxon signed-rank test, as implemented in Bottleneck version 1.2.02 [43]). Most microsatellites fit a two-phase model of mutation (TPM) better than a strict stepwise mutation model (SMM) or infinite alleles model (IAM) [44]. However, microsatellites with imperfect repeat motifs are more likely to evolve under the IAM model [45]. As we found no strong a priori reason to select or reject one model over another, we ran Bottleneck under all three mutational models. We used a TPM model with the default settings of 30% variation from the IAM model and 70% from the SMM model.

The levels of genetic differentiation between pairs of populations were estimated using pairwise *F*_ST_ values [46] computed with 10000 permutations in Arlequin [37]. The AMOVA [47] was performed using the Arlequin program to calculate the variance among and within groups and within populations. Significance for AMOVA analysis was ascertained using 10000 permutations. We used sequential Bonferroni correction for part tests involving multiple comparisons [48].

To assess population genetic structure, we used a Bayesian model-based clustering analysis with Structure 2.3.3 [49], [50]. We specified an initial range of potential genotype clusters (K) from 1 to 10 under the admixed model and the assumption of correlated allele frequencies among populations. For each value of K, 10 runs were performed with 100000 iterations discarded as burn-in followed by an additional 1 million iterations. The most probable number of K in the data was detected both by comparing the log probability of the data lnP (D) for each value of K across all 10 runs of Structure and by examining the standardized second-order change of lnP (D), *Δ*K [51]. For the selected values of K, the software CLUMPP v1.1.2 [52] was used to align cluster membership coefficients from the 10 replicate cluster analyses using the Greedy algorithm with 10000 random input orders, the results were graphically displayed with DISTRUCT 1.1 [53].

A Mantel test for isolation by distance, as revealed by a correlation between pairwise linearized genetic and log-geographic distances (Euclidean) [54], was performed using IBDWS 3.16 [55], with significant level evaluated based on 10000 permutations. IBDWS uses a Reduced Major Axis (RMA) regression to estimate the slope and intercept of the isolation by distance relationship.

Asymmetric pairwise measures of recent gene flow were estimated using two different genetic approaches: (i) assignment tests and detection of first generation migrants for each population implemented in Geneclass 2 [56], and (ii) a Bayesian approach implemented in BayesAss+ to estimate recent migration rates [57]. The Geneclass tests were performed using a partially Bayesian method [58] and Monte-Carlo resampling algorithm of Paetkau et al. [59] with 10000 simulated individuals and type I error of 0.01. Otherwise, L-home likelihood computation, which is the most appropriate statistics when all potential source populations have not been sampled, was used to detect first generation migrants. BayesAss+ was implemented with 3000000 MCMC iterations, with a burn-in of 1000000 iterations to allow the chain to reach stationarity, a sampling frequency of 2000, and delta values were adjusted to ensure that 40–60% of the total changes were accepted. Only migration estimates whose confidence intervals did not overlap with the confidence limits of a simulated distribution with no information content were considered reliable (see BayesAss+ manual).

## Results

### Sequence diversity and phylogenetic pattern

An alignment of a fragment of the COI gene (571 bp) from 506 *F. occidentalis* individuals from 14 populations across China revealed five haplotypes (Hap 1, 2, 3, 4 and 5, GenBank accession numbers are JN790696, JN790697, JN790698, JN790699 and JN790700, Table 3). The sequences had 22 polymorphic sites, of which 21 were parsimony informative. The number of haplotypes per population ranged from 2 to 4 (Table 3). The haplotype diversity (*H*_d_) ranged between 0.172 and 0.656 with the lowest in HRB (The locations and abbreviations of the 14 populations are shown in Table 1) and highest in TA (Table 3, Figure 1). The most common haplotype (Hap1) was found in 316 individuals representing all 14 populations. Hap2 was found in 85 individuals from 10 populations and Hap3 was found in 92 individuals from 12 populations. Interestingly, a haplotype (Hap5) that has not been found in other studies was found in one of the populations from southwestern China, DL, which had high *H*_d_. Hap4, which was found in 12 individuals across 7 populations, was separated by at least 19 mutations from the other four haplotypes (Figure 1B) and formed a distinct lineage. Rugman-Jones et al. provided strong evidence that this lineage (Hap4 in this study) could represent a cryptic species, western flower thripsL (WFTL). The other lineage (Hap1, 2, 3, and 5 in this study) was referred to as western flower thripsG (WFTG) [20]. When compared to the results of Brunner & Frey [19], Hap1 and Hap2 correspond to the hot/dry (HD) ecotype and Hap4 corresponds to the cool/moist (CM) ecotype. One haplotype (Hap3) was not discovered in Brunner & Frey [19]. Furthermore, the sequences of 28SD2 were identical across all the 92 randomly selected individuals representing the five mitochondrial haplotypes identified in China.

**Table 3:** Basic indices calculated using COI gene and ten microsatellites and haplotype distribution in Chinese populations.

POP	mtDNA									microsatellite				
	*N*_h_	Hap1	Hap2	Hap3	Hap4	Hap5	*H*_d_ (±SD)	k	π (±SD)	*A*_P_	*A*_R_	*H*_O_	*uH*_E_	*H*_S_
BJ	3	29	15	4	0	0	0.542 (0.052)	0.595	0.00104 (0.00013)	0	4.517	0.531	0.727	0.729
DH	3	14	0	8	2	0	0.565 (0.071)	3.493	0.00612 (0.00312)	2	4.512	0.570	0.718	0.721
GY	3	17	1	12	0	0	0.536 (0.048)	0.563	0.00099 (0.00012)	0	4.361	0.586	0.689	0.691
JQ	2	30	0	5	0	0	0.252 (0.085)	0.252	0.00044 (0.00015)	5	4.728	0.654	0.750	0.751
HRB	3	40	1	3	0	0	0.172 (0.074)	0.175	0.00031 (0.00013)	2	4.707	0.613	0.733	0.734
QHD	3	40	3	4	0	0	0.270 (0.081)	0.281	0.00049 (0.00015)	1	4.817	0.541	0.722	0.724
CC	2	8	0	0	2	0	0.356 (0.159)	6.756	0.01183 (0.00529)	0	5.027	0.626	0.770	0.778
SY	4	29	13	4	1	0	0.547 (0.060)	1.377	0.00241 (0.00133)	1	4.349	0.593	0.720	0.721
QTX	2	4	0	0	3	0	0.571 (0.119)	10.857	0.01901 (0.00398)	0	3.776	0.506	0.621	0.633
QD	4	27	9	10	1	0	0.600 (0.057)	1.467	0.00257 (0.00133)	1	4.549	0.610	0.709	0.710
TA	3	19	12	10	0	0	0.656 (0.035)	0.802	0.00141 (0.00013)	1	4.553	0.562	0.707	0.708
BS	4	19	21	6	2	0	0.648 (0.037)	2.276	0.00399 (0.00175)	1	4.716	0.627	0.737	0.738
DL	4	13	3	13	0	1	0.634 (0.049)	0.761	0.00133 (0.00018)	0	5.191	0.595	0.764	0.767
KM	4	27	7	13	1	0	0.601 (0.053)	1.449	0.00254 (0.00131)	2	4.933	0.614	0.759	0.760
Total		316	85	92	12	1								
Mean							0.496				4.624	0.588	0.723	0.726

*N*_h_, number of haplotypes; *H*_d_, haplotype diversity; k, average number of nucleotide differences; π, nucleotide diversity; *A*_P_, number of private alleles; *A*_R_, allelic richness; *H*_O_, observed heterozygosity; *uH*_E_, unbiased expected heterozygosity; *H*_S_, gene diversity.

### Microsatellite markers

**Figure 2:**
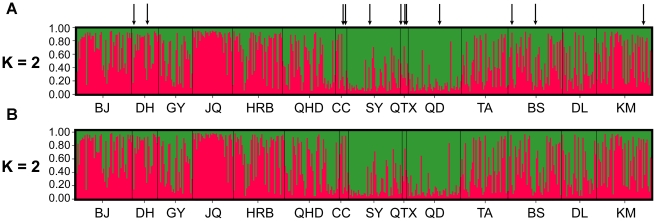
Bayesian clustering analysis using STRUCTURE indicating the presence of two clusters. Each individual is represented by a vertical bar displaying membership coefficients to each genetic cluster. (A) Analysis based on the whole datasets, the arrows point to the 12 WFTL individuals. (B) Analysis based only on WFTG individuals.

Coexistence of two different forms [19], [20] in our sample might influence the results and conclusions of our study. Thus, we performed our analysis on two different datasets. One included both forms while the other included only WFTG/HD. The results revealed that there was excellent concordance between these two datasets (Table 3–5, Table S2, S3, S4, Figure 2). This might be due to the low number of the WFTL/CM (n = 12) or the occurrence of hybridization between the two forms in China (see Discussion). Therefore we present the results of the whole datasets below.

**Table 4 pone-0034567-t004:** Pairwise *F_ST_* matrix obtained using 10 microsatellite loci.

	BJ	DH	GY	JQ	HRB	QHD	CC	SY	QTX	QD	TA	BS	DL
DH	**0.043**												
GY	**0.057**	**0.061**											
JQ	**0.072**	**0.086**	**0.064**										
HRB	**0.025**	**0.040**	**0.043**	**0.058**									
QHD	**0.033**	**0.043**	**0.039**	**0.063**	**0.020**								
CC	0.018	0.007	0.016	**0.047**	0.012	0.015							
SY	**0.066**	**0.060**	**0.063**	**0.084**	**0.052**	**0.029**	0.026						
QTX	0.043	**0.087**	**0.067**	**0.090**	0.044	0.043	0.047	**0.078**					
QD	**0.067**	**0.053**	**0.060**	**0.097**	**0.062**	**0.055**	0.007	**0.057**	**0.064**				
TA	**0.053**	**0.059**	**0.058**	**0.079**	**0.049**	**0.052**	0.017	**0.069**	**0.060**	**0.038**			
BS	**0.022**	**0.024**	**0.028**	**0.063**	**0.018**	**0.015**	−0.008	**0.033**	**0.051**	**0.033**	**0.042**		
DL	**0.028**	**0.036**	**0.023**	**0.060**	**0.028**	0.010	−0.006	**0.034**	0.041	**0.029**	**0.033**	0.008	
KM	**0.025**	0.019	**0.037**	**0.058**	**0.014**	**0.017**	−0.007	**0.040**	**0.044**	**0.043**	**0.031**	0.013	0.012

Bold indicates significant values after Bonferroni correction (P = 0.05).

**Table 5:** Results of assignment test and detection of first generation migrants (F0), with source populations list by column and recipient populations by row.

	BJ	DH	GY	JQ	HRB	QHD	SY	QD	TA	BS	DL	KM
BJ	**28**	1				1			1	2	6 (1)	8 (1)
DH		**20**									2	2
GY			**20**					1			8 (1)	1
JQ				**34**						**(1)**	1 (1)	
HRB	1				**29**	3 (1)		1 (1)			6	4
QHD		1			1	**29**	1			2	10 (3)	3 (1)
SY					1	2	**29**	1 (1)		1	10 (1)	2
QD							1	**38**		1	5 (1)	2
TA		2							**28**		9 (1)	2
BS						3				**29**	13 (2)	3
DL									1	**(3)**	**28**	1
KM		1					1			1 **(2**)	5 (1)	**40**

Populations with sample size of ≤10 individuals were not included.

### Intrapopulation genetic diversity

All the newly developed microsatellite markers proved to be polymorphic (Table 2). The number of alleles per locus ranged from 5 to 23 and *H*_O_ ranged from 0.085 to 1.000 (Table 2 and Table S1). A total of 506 individuals representing the 14 introduced populations in China were genotyped with 10 microsatellite markers. Significant linkage disequilibrium was present in 28 out of a total of 630 tests (α = 0.05), but just two (WFT04 and WFT07 in QD; WFT02 and FOCC75 in DL) were significant after sequential Bonferroni corrections. The linkage disequilibrium was possibly due to population structures rather than physical linkage because significant allelic association was not consistently restricted to certain pairs of loci in all populations analyzed. After sequential Bonferroni corrections for multiple comparisons [48], all populations except CC and QTX with small sample sizes deviated significantly from Hardy-Weinberg equilibrium (HWE). Fisher's exact tests showed that 28 of the 140 locus/population combinations deviated significantly from HWE. Of these, 26 cases were concentrated in four loci (WFT03, WFT04, WFT07 and FOCC75). In all cases, the deviations were associated with a significant positive *F*_IS_ value.

The allelic richness (*A*_R_) based on all 10 loci was strongly correlated with the *A*_R_ value based on 6 loci without the 4 problematic loci (Spearman r = 0.903, P<0.001). Similar patterns were observed for the unbiased expected heterozygosity (*uH*_E_; Spearman r = 0.719, P = 0.002). Thus, all 10 loci were used in the subsequent analysis. The genetic diversity indices *uH*_E_ and *A*_R_ were not statistically significant across all 14 populations (one-way ANOVA, F_13, 126_ = 0.401 and 0.630, P = 0.967 and 0.825, respectively). The average allelic richness (*A*_R_) ranged from 3.776 to 5.191 alleles in QTX and DL localities, respectively. *uH*_E_ ranged from 0.621 to 0.770 in QTX and CC, respectively (Table 3). When all the populations were partitioned into two groups based on the pairwise *F*_ST_ analysis below, the group that includes the three homogenous populations (BS, DL, KM) in southwestern China exhibited a statistically significant higher allelic richness (*A*_R_ = 4.946 vs 4.536; P = 0.027), gene diversity (*H*_S_ = 0.753 vs 0.722; P = 0.019) and a lower *F*_ST_ (*F*_ST_ = 0.010 vs 0.051; P = 0.014) than the other group which includes the other populations. The same results were obtained when the two small populations (CC, QTX) were excluded from the comparative analysis.

The Bottleneck test revealed that 7 of 14 populations had a statistically significant excess of heterozygotes under the IAM, suggesting these populations might undergo a recent genetic bottleneck (Table S5). Additionally, the test was non-significant for all populations under the TPM and SMM models (Table S5).

### Interpopulation genetic differentiation

The global *F*_ST_ value, calculated across all populations and loci within China, was 0.043 (P<0.001) indicating a low but significant population differentiation in China. When considering all the populations, 70 of the 91 pairwise comparisons tested were significantly different from zero (Table 4). QTX and CC were not significantly different from most other populations, possibly due to the lack of sufficient power to detect a significant difference when unbalanced sampling schemes are used [60]. Excluding these two populations, there are 5 population comparisons with nonsignificant *F*_ST_ values: BS versus DL and KM; DL versus QHD and KM; KM versus DH. Interestingly, three geographically close populations in southwestern China (BS, DL and KM) appeared homogenous, with no significant differentiation following the use of Bonferroni correction, suggesting the occurrence of extensive gene flow among these three populations. Additionally, JQ seems to be an isolated population since it was significantly differentiated from the other populations (mean *F*_ST_ = 0.079) (Table 4).

**Figure 3:**
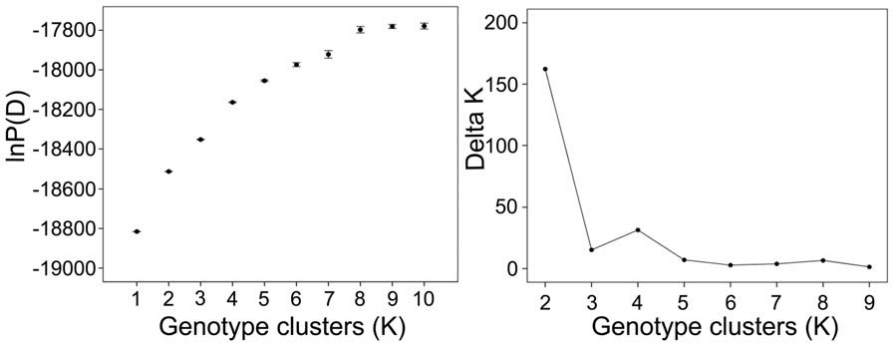
Inference of the number of genetic clusters (K) from STRUCTURE simulations for Chinese *Frankliniella occidentalis* populations. The likelihood of the data given K [ln P(D); left] and ΔK ([51]; right) are plotted against the number of genetic clusters (K). Error bars represent standard deviations over ten runs.

The Bayesian clustering analysis for *F. occidentalis* revealed that K = 2 was the best fit of the data for the 14 populations in China (Figure 3). BJ, DH, JQ and HRB formed one genotype cluster and SY, QTX, QD were assigned to the other cluster with a slightly higher membership coefficient (Q>0.6). Individuals from the other populations are a mixture of individuals that did not appear to belong to any of the two genotype clusters (0.40<Q<0.60). More strikingly, the WFTL/CM individuals cannot be distinguished from the WFTG/HD individuals (Figure 2). It was also supported by the AMOVA analysis which revealed no genetic differentiation between the two forms at microsatellite loci (Table S6).

The AMOVA analysis revealed that more than 88% and 95% of variation was attributed to among individuals within populations using mitochondrial and microsatellite data, respectively, and only a small portion of the variation was attributed to among populations within groups and among groups. Furthermore, the microsatellite data did not reveal any significant variation between the putative source populations and the other populations but it did reveal significant variation between the clusters inferred by STRUCTURE. Additionally, no significant variation was found among groups using the mitochondrial data (Table S6).

**Figure 4:**
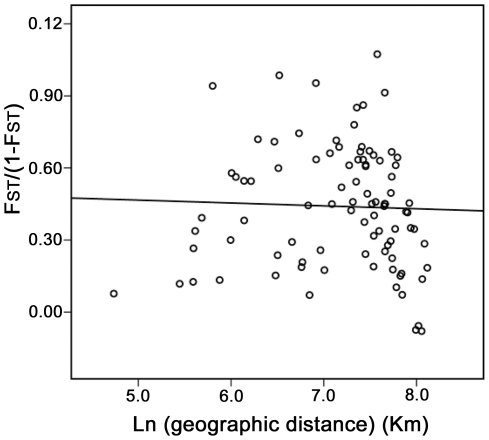
Correlation between pairwise linearzed *F*_ST_ values and the logarithm of geographic distance in Chinese populations of *Frankliniella occidentalis.* Reduced Major Axis (RMA) regression line is shown.

If the dispersal of *F. occidentalis* is limited by distance, genetic and geographical distances should be positively correlated, producing a pattern of isolation-by-distance. On the contrary, the isolation by distance correlation was non-significant and slightly negative (Z = 28.655, r = −0.035, P = 0.349; Figure 4), suggesting a possible anthropogenic influence on the spread of the *F. occidentalis* in China.

### Individual assignment tests

The partial Bayesian method computed with GeneClass revealed high overall assignment success, approximately 99.6% (487 of 489 individuals were definitively classified at a P value of 0.05). The majority of putative migrant genotypes (110/135) were restricted to southwestern China and the migration events were highly directional, from the Southwest to other parts of China. GeneClass also identified 23 individuals as potentially first-generation (F_0_) migrants, of which 20 migrated from the three southwestern populations to almost all of the other populations (Table 5). Together, the GeneClass results suggest that a directional spread occurred from the three southwestern populations to other parts of China. The BayesAss+ analysis revealed that our data set did not contain enough information to suitably estimate migration since the confidence intervals recovered from the data set always overlapped those obtained from the null hypothesis.

## Discussion

This study constitutes the first attempt to understand the pattern of genetic variability in *F. occidentalis* as a consequence of its introduction and expansion in China. Given the short time span between detection of the invasion and observations of this study (10 years), our results shed light on the dynamic aspects of the invasion process soon after the introduction.

### Genetic diversity and population differentiation

Overall, our genetic diversity estimates for *F. occidentalis* in China are lower than those reported in the native regions based on mitochondrial and microsatellite DNA markers [19]. For example, the mitochondrial genetic diversity estimates for the 14 invasive populations in China (*N*_h_ = 5, *H*_d_ = 0.549) were much lower than those reported in the 12 native populations in the United States (*N*_h_ = 30, *H*_d_ = 0.897; [19]). Consistent with the mtDNA data, the microsatellite genetic diversity indices (*uH*_E_ = 0.723 and *H*_S_ = 0.726) in the 14 invasive populations were lower than those in the United States (*uH*_E_ = 0.855 and *H*_S_ = 0.853) [19]. Moreover, the genetic diversity estimates (*N*_h_ and *uH*_E_) for *F. occidentalis* in China was comparable to those reported for populations of fire ant *Solenopsis invicta* in newly invaded areas in China, Australia, New Zealand and the Caribbean (*N*_h_ = 3; [61]) and *Bactrocera invadens* in Africa (*H*_E_ = 0.56, computed over 11 microsatellites; [62]). As expected, the bottleneck signature in China is more pronounced in the mitochondrial genome, which is subject to stronger genetic drift than the nuclear genome because of its maternal and haploid mode of inheritance and reduced effective population sizes [32], [63], [64]. However, due to the use of different microsatellite markers in our study and the studies of Brunner & Frey [19], it was not possible to estimate the reduction in nuclear diversity between the introduced populations from China and populations from the native range.

Furthermore, the reduction in genetic diversity possibly due to bottlenecks as revealed by the Bottleneck analysis with several populations of *F. occidentalis* across China showed recent reductions in population sizes under the IAM. However, under the TPM and SMM models, none of these populations exhibited a heterozygosity excess, which may be because heterozygosity excess observed in populations that have declined in size is a transient feature, expected to last only a few generations [65]. In many cases, we also do not know whether the populations we examined were within this small population period. Additionally, the high gene flow among the Chinese populations might obscure the genetic effects of a bottleneck via the introduction of rare alleles, especially under the more conserved SMM model. Thus, although the IAM can occasionally detect genetic bottlenecks erroneously [65], the above analysis coupled with the low genetic diversity suggested that slight genetic bottlenecks might take place in these populations. The bottlenecks could be due to a small founding population or few introduction events. The invasion success of this species does not appear to depend on high levels of genetic variation, although we did not measure the variation of adaptive traits. Indeed, most successful invasive insect species show a reduction in genetic diversity from the native to invaded areas [4], [6], [66], [67]. The successful invasion of *F. occidentalis* in China is probably due to its biological attributes together with the existence of numerous suitable habitats and climates across China [68]. The minute size, cryptic behavior, egg deposition inside plant tissue and polyphagous nature (feeding on over 250 different plants in 62 different families [69]), make detection difficult and facilitate the invasion of *F. occidentalis* to new environments. In addition, because of its high fecundity (average total lifetime fecundities exceeding 200 progeny per female [70]), high resistance to many pesticides [71], high population growth potential [72], *F. occidentalis* can easily become established in new areas. The haplodiploid sex determination of *F. occidentalis* makes it relatively resistant to the detrimental effects of inbreeding and allows to rapidly adapt to new suitable environments [17].

All populations of *F. occidentalis* showed significant deviation from HWE due to heterozygosity deficiency that could arise from recurrent inbreeding, subpopulation structure (i.e. Wahlund effect) and/or null alleles. Brunner & Frey also found a significant deviation from HWE of *F. occidentalis* within two different habitats in its native area [19]. Strong inbreeding may be the main factor contributing to the departure from HWE (0.124<*F*_IS_<0.276 with a mean of 0.188), since a female thrips could survive long enough to mate with her own haploid male progeny because of their special haplodiploid sex determination, potentially long adult lifespan and rapid immature development rate [17], [73]. An analogous situation was observed in the invasive haplodiploid palm-seed borer *Coccotrypes dactyliperda* whose principle mating strategy was inbreeding with an average inbreeding coefficient of *F*_IS_ = 0.27 within populations in California [74]. Three species of solitary gall thrips (*Kladothrips xiphius*, *K. arotrum*, *K. antennatus*) were also highly inbred (*F*_IS_ = 0.54–0.68; [75]). A Wahlund effect is unlikely given that there is no subpopulation structure in most populations in China. Null alleles could not have major impacts on the heterozygote deficiency due to the highly successful PCR amplification rate (>96.8% for each locus across all populations). However, we could not rely on software programs that test for null alleles because they assume random mating.

Analysis of *F. occidentalis* population structure in China based on *F*_ST_ and AMOVA analysis revealed weak but significant differentiation (global *F*_ST_ = 0.043, p<0.001) which is comparable to the differentiation level of an invasive fruit fly pest *B. invadens* across its introduced range in Africa (0.015<*F*_ST_<0.129; [62]). The low differentiation and the absence of isolation by distance may be largely due to the high gene flow, especially over long distances, which should homogenize gene frequencies over populations within China. Anthropogenic transport is the most likely explanation for the large-scale dispersion of *F. occidentalis*, since it is a weak flier and other localities are far away (110–3300 km) from the source populations in southwestern China. This is especially the case for DH, which is separated by desert and xeric grasslands from other desert-edge localities where more host plants could survive. The main means of spreading of *F. occidentalis* is the movement of floricultural and horticultural products [21], and this is probably also the case in China, although other means such as wind currents [76] cannot be ruled out.

Furthermore, JQ with more private alleles (*N*p = 5) seems to be an isolated population with significant and much higher *F*_ST_ compared with other populations. Its isolation is also supported by the Bayesian analysis. The isolation of JQ might be the result of genetic drift, natural selection or a separate introduction event(s). Multiple introductions seem unlikely given JQ has only two mitochondrial haplotypes which also exist in most populations across China. The presence of a greater number of nuclear private alleles in JQ is probably due to the low sample number in the founder populations and high allele number of microsatellites.

### Hybridization between the two forms of *F. occidentalis*

Our mtDNA results (but not the microsatellites) confirmed the presence of two previously described forms of *F. occidentalis* in China. The two forms were suggested to be different cryptic species (WFTG and WFTL) [19] or different ecotypes (HD and CM) [20]. One form (WFTG/HD) was observed at extremely higher frequency (494/506) than the other (WFTL/CM) (12/506). At the global scale, WFTG/HD established in almost every continent, but WFTL/CM established only in New Zealand outside its native range [20]. The invasive pest *Bemisia tabaci* also has several biotypes with different degrees of invasive success [77], [78]. The difference in apparent distribution and abundance of these two cryptic species in the introduced range could result from their different biology attributes or selection favoring some traits related to climate, natural enemies and/or insecticide resistance. However, an unequal number of importations of these two cryptic species cannot be ruled out. Brunner & Frey have shown that the ecological niche adaptation may be among the key factors determining the astonishing invasion potential of *F. occidentalis* [19]. In addition, although there were no direct comparisons between these two cryptic species, several studies might unwittingly show that WFTG/HD exhibits higher fecundity and higher resistance level to insecticides than WFTL/CM [20], [79], [80].

In China, these two forms were about 3.3% divergent in terms of their mtDNA, but they cannot be distinguished by microsatellite markers. Moreover, the 28SD2 sequences of these two cryptic species in China were identical to each other and corresponded to the WFTG/HD which reported in its native range [20]. It is unlikely that the observed mitochondrial-nuclear discordance is the result of incomplete lineage sorting since significant differences were detected between these two cryptic species at the same nuclear and mitochondrial loci in its native range [20]. The analyses above suggested that hybridization between these two forms might produce such substantial mitochondrial-nuclear discordance. If these two forms are different cryptic species, the Chinese WFTL individuals were probably derived from unidirectional introgression of the WFTL mitochondrial genome into the WFTG nuclear background through hybridization. Extensive mitochondrial introgression have been broadly documented in insects [81], [82] and other organisms, including crustacean, fish, and mammals [83]–[85]. Introgressive hybridization was observed between the two cryptic species in China, but reproductive isolation in its native region was evidenced by Rugman-Jones et al. [20]. One explanation for these two distinct phenomena is that WFTG and WFTL are sympatric in China, but they are allopatric in their native range except several populations [19]. Another explanation is that the rare WFTL individuals coexisted with more abundant WFTG individuals (discussed above) in China, but approximately equal abundance in their native populations where they were sympatric [20]. This explanation is consistent with the unidirectional hybridization hypothesis that female mate discrimination should encourage hybrid reproduction between females of a rare species and males of a common one [86]. Chan & Levin [87] also demonstrated that certain models of frequency-dependent prezygotic reproductive barriers allow for very rapid biased introgression of maternally inherited genomes. This phenomenon has been reported in several studies [83], [86]. In addition, we cannot completely rule out the possibility that the introgressive hybridization of the two *F. occidentalis* cryptic species in China arises from other factors, such as natural selection, change of habitat. Alternatively, if these two forms are different ecotypes, it was possible for hybridization to occur in China where they were sympatric, because they are not completely reproductive isolated [19]. Since hybridization has taken place in China, further studies should be conducted on whether the fitness of these hybrids is enhanced or reduced. This knowledge is crucial for the understanding of the evolutionary impact of invasive species and the integrated control of this pest.

### Migration patterns in China

The *F. occidentalis* neighboring populations BS, DL and KM are genetically similar and form a single population and differ little from several other populations with which they share co-ancestry. Furthermore, these three populations have an asymmetric migration towards other populations and have a slighter higher microsatellite genetic diversity than do other populations. Consistent with the nuclear microsatellite variation, these three populations have a higher mitochondrial DNA diversity. Together, these analyses provide compelling evidence that the invasion started in southwestern China (BS, DL and KM), where *F. occidentalis* was initially found. This hypothesis is supported by the fact that KM is a center for floriculture production and transportation and a major centre for international imports of plants in China [25], where thrips are frequently intercepted. Thus, KM is probably the port of entry of *F. occidentalis*. This appears to be another case of the Bridgehead effect [9], which was predicted to be common [88] and has been demonstrated by the movement of several invasive insects. The fire ant *Solenopsis invicta*, which is native to South America, was inadvertently introduced into the southern United States, where it formed the bridgehead populations that act as a source of the California populations and other populations in China, Australia, New Zealand and the Caribbean [61]. The western corn rootworm, *Diabrotica virgifera virgifera*, which is native to Mexico, established a bridgehead population in the United States for its later invasion of Europe [10], [89]. The invasion of Europe, South America and Africa by *Harmonia axyridis* also followed the establishment of a bridgehead population in eastern North America [9].

### Implications for management

New knowledge on the genetic diversity and population structure of *F. occidentalis* in China as revealed in this study can help to improve management strategies. Strict trade quarantines and local eradication should be imposed to prevent further introduction and spread of the putative three source populations of *F. occidentalis* because of the high rates of gene flow from the three southwestern populations to the rest of the populations in China. Preventing contaminated plants from the putative source populations from being transported and population suppression or local eradication are crucial to eradicating this pest in other areas. To eradicate *F. occidentalis* from an area, it is necessary to reduce the population by various means, such as by using insecticides and chemical attractants, to a level below the Allee threshold or to apply biological controls to increase the Allee threshold [13].

## Supporting Information

Table S1 Genetic diversity at ten microsatellite loci in 14 *Frankliniella occidentalis* populations in China.
(DOC)

Table S2 Basic indices calculated using COI gene and ten microsatellites based only on WFTG individuals and haplotype distribution in Chinese populations.
(DOC)

Table S3 Pairwise *F_ST_* matrix obtained using 10 microsatellite loci. All WFTL individuals were omitted from the respective population and were treated as a single population.
(DOC)

Table S4 Results of assignment test and detection of first generation migrants (F0) based on WFTG individuals, with source populations list by column and recipient populations by row. Populations with sample size of ≤10 individuals were not included.
(DOC)

Table S5 Within-population tests for heterozygosity excess P-values.
(DOC)

Table S6 Results of AMOVA test on mitochondrial and microsatellite markers.
(DOC)
