# Supplementary material for: Invasion Genetics of the Western Flower Thrips in China: Evidence for Genetic Bottleneck, Hybridization and Bridgehead Effect
Source: PLoS One. 2012 Apr 3;7(4):e34567. doi: 10.1371/journal.pone.0034567 (PMC3317996; doi:10.1371/journal.pone.0034567)
Supplement: Table S1 — Genetic diversity at ten microsatellite loci in 14 Frankliniella occidentalis populations in China. (DOC) [file pone.0034567.s001.doc]

**Table S1**. Genetic diversity at ten microsatellite loci in 14 *Frankliniella occidentalis* populations in China.

| **Populations** | **Index** | **Microsatellites** | | | | | | | | | | |
| --- | --- | --- | --- | --- | --- | --- | --- | --- | --- | --- | --- | --- |
|  |  | **WFT01** | **WFT02** | **WFT03** | **WFT04** | **WFT05** | **FOCC125** | **FOCC75** | **WFT06** | **WFT07** | **WFT08** | **Mean** |
| Beijing | *N* | 48 | 48 | 48 | 48 | 48 | 47 | 47 | 48 | 46 | 48 |  |
|  | *A*/*A*_R_ | 9/5.371 | 6/4.353 | 3/1.958 | 10/5.721 | 6/4.215 | 7/4.328 | 12/5.700 | 14/5.166 | 12/5.871 | 5/2.490 | 8.4/4.517 |
|  | *H*_O_ | 0.8125 | 0.5833 | 0.1875 | 0.4583 | 0.6667 | 0.5319 | 0.5957 | 0.8125 | 0.2826 | 0.3750 | 0.5306 |
|  | *uH*_E_ | 0.8478 | 0.7809 | 0.2980 | 0.8535 | 0.7294 | 0.7108 | 0.8474 | 0.8002 | 0.8493 | 0.5540 | 0.7271 |
|  | *H*_S_ | 0.848 | 0.783 | 0.299 | 0.858 | 0.73 | 0.713 | 0.85 | 0.8 | 0.856 | 0.556 | 0.7293 |
|  | *F*_IS_ | 0.0421 | 0.2550 | 0.3733 | 0.4656 | 0.0868 | 0.2537 | 0.2992 | -0.0155 | 0.6697 | 0.3254 | 0.2755 |
|  | *r* | 0.017 | 0.1218 | 0.1442 | 0.2239 | 0.0277 | 0.1258 | 0.143 | -0.0155 | 0.3183 | 0.1477 |  |
|  | P_HWE_ | 0.2953 | 0.0152 | 0.0255 | 0.0000 | 0.6127 | 0.0031 | 0.0000 | 0.5101 | 0.0000 | 0.0051 |  |
| Dunhuang | *N* | 24 | 24 | 24 | 24 | 24 | 24 | 23 | 22 | 23 | 24 |  |
|  | *A*/*A*_R_ | 9/5.321 | 6/4.320 | 4/2.427 | 11/5.692 | 7/4.487 | 7/3.317 | 11/6.233 | 9/4.724 | 10/6.229 | 3/2.372 | 7.7/4.512 |
|  | *H*_O_ | 0.6667 | 0.7083 | 0.2083 | 0.3333 | 0.7083 | 0.5833 | 0.5217 | 0.8636 | 0.5652 | 0.5417 | 0.5701 |
|  | *uH*_E_ | 0.8227 | 0.7793 | 0.3316 | 0.8440 | 0.7358 | 0.5931 | 0.8860 | 0.7674 | 0.8870 | 0.5284 | 0.7175 |
|  | *H*_S_ | 0.826 | 0.781 | 0.334 | 0.855 | 0.736 | 0.593 | 0.894 | 0.765 | 0.894 | 0.528 | 0.7206 |
|  | *F*_IS_ | 0.1930 | 0.0928 | 0.3767 | 0.6102 | 0.0381 | 0.0168 | 0.4166 | -0.1287 | 0.3680 | -0.0257 | 0.1958 |
|  | *r* | 0.0787 | 0.0316 | 0.1808 | 0.2929 | 0.0203 | -0.0046 | 0.1963 | -0.0909 | 0.1697 | -0.022 |  |
|  | P_HWE_ | 0.0763 | 0.0251 | 0.0171 | 0.0000 | 0.8492 | 0.5521 | 0.0000 | 0.5341 | 0.0000 | 1.0000 |  |
| Guiyang | *N* | 30 | 30 | 30 | 30 | 30 | 30 | 28 | 29 | 28 | 30 |  |
|  | *A*/*A*_R_ | 7/4.565 | 7/3.279 | 2/1.612 | 8/5.178 | 7/4.899 | 9/5.162 | 10/4.862 | 12/5.570 | 11/5.580 | 4/2.903 | 7.7/4.361 |
|  | *H*_O_ | 0.7667 | 0.5000 | 0.1000 | 0.5000 | 0.8000 | 0.7333 | 0.3571 | 0.8276 | 0.6786 | 0.6000 | 0.5863 |
|  | *uH*_E_ | 0.7932 | 0.4757 | 0.1554 | 0.8299 | 0.8136 | 0.8215 | 0.7812 | 0.8361 | 0.8234 | 0.5565 | 0.6886 |
|  | *H*_S_ | 0.794 | 0.475 | 0.156 | 0.836 | 0.814 | 0.823 | 0.789 | 0.836 | 0.826 | 0.556 | 0.6905 |
|  | *F*_IS_ | 0.0340 | -0.0520 | 0.3603 | 0.4017 | 0.0169 | 0.1089 | 0.5474 | 0.0103 | 0.1785 | -0.0796 | 0.1526 |
|  | *r* | 0.0082 | -0.0353 | 0.1135 | 0.1854 | 0.0003 | 0.0389 | 0.2582 | -0.0062 | 0.0766 | -0.0425 |  |
|  | P_HWE_ | 0.5437 | 0.6216 | 0.1623 | 0.0002 | 0.3869 | 0.0456 | 0.0000 | 0.5034 | 0.3282 | 0.9161 |  |
| Jiuquan | *N* | 35 | 35 | 35 | 35 | 35 | 35 | 35 | 35 | 33 | 35 |  |
|  | *A*/*A*_R_ | 11/5.319 | 7/4.414 | 4/2.834 | 11/5.738 | 7/3.998 | 9/4.995 | 11/5.942 | 13/6.366 | 9/4.873 | 3/2.802 | 8.5/4.728 |
|  | *H*_O_ | 0.7143 | 0.7143 | 0.4571 | 0.6286 | 0.6571 | 0.8000 | 0.7714 | 0.8000 | 0.4546 | 0.5429 | 0.6540 |
|  | *uH*_E_ | 0.8070 | 0.7536 | 0.4899 | 0.8550 | 0.6899 | 0.8211 | 0.8526 | 0.8899 | 0.7618 | 0.5743 | 0.7495 |
|  | *H*_S_ | 0.808 | 0.754 | 0.49 | 0.858 | 0.69 | 0.821 | 0.854 | 0.891 | 0.767 | 0.575 | 0.7508 |
|  | *F*_IS_ | 0.1164 | 0.0529 | 0.0677 | 0.2677 | 0.0481 | 0.0261 | 0.0965 | 0.1023 | 0.4070 | 0.0556 | 0.124 |
|  | *r* | 0.0542 | 0.0144 | 0.0007 | 0.1264 | 0.0257 | 0.005 | 0.0457 | 0.0427 | 0.1998 | 0.0225 |  |
|  | P_HWE_ | 0.0874 | 0.0133 | 0.3588 | 0.0914 | 0.1933 | 0.9300 | 0.0654 | 0.1309 | 0.0000 | 0.0012 |  |
| Harbin | *N* | 44 | 44 | 44 | 44 | 44 | 44 | 43 | 44 | 44 | 44 |  |
|  | *A*/*A*_R_ | 8/4.929 | 7/4.147 | 4/2.279 | 9/5.161 | 7/4.517 | 9/4.964 | 14/6.055 | 13/5.207 | 16/6.848 | 4/2.967 | 9.1/4.707 |
|  | *H*_O_ | 0.8636 | 0.6364 | 0.2273 | 0.2273 | 0.7727 | 0.5909 | 0.6977 | 0.8636 | 0.7046 | 0.5455 | 0.6129 |
|  | *uH*_E_ | 0.8169 | 0.6800 | 0.3169 | 0.8028 | 0.7435 | 0.7816 | 0.8473 | 0.8077 | 0.9054 | 0.6262 | 0.7328 |
|  | *H*_S_ | 0.816 | 0.68 | 0.318 | 0.809 | 0.743 | 0.784 | 0.849 | 0.807 | 0.908 | 0.627 | 0.7341 |
|  | *F*_IS_ | -0.0579 | 0.0649 | 0.2851 | 0.7192 | -0.0398 | 0.2461 | 0.1784 | -0.0701 | 0.2239 | 0.1302 | 0.168 |
|  | *r* | -0.0353 | 0.0313 | 0.108 | 0.3396 | -0.0338 | 0.1027 | 0.0807 | -0.0422 | 0.1087 | 0.0642 |  |
|  | P_HWE_ | 0.9834 | 0.6224 | 0.0892 | 0.0000 | 0.9834 | 0.0009 | 0.0127 | 0.2982 | 0.0175 | 0.2520 |  |
| Qinhuangdao | *N* | 47 | 47 | 47 | 46 | 46 | 47 | 46 | 47 | 46 | 47 |  |
|  | *A*/*A*_R_ | 12/6.058 | 7/4.344 | 3/1.703 | 11/5.784 | 8/4.184 | 12/4.830 | 14/5.802 | 13/5.797 | 16/6.893 | 4/2.778 | 10/4.817 |
|  | *H*_O_ | 0.8723 | 0.7447 | 0.0851 | 0.4130 | 0.5435 | 0.4681 | 0.6739 | 0.7234 | 0.4565 | 0.4255 | 0.5406 |
|  | *uH*_E_ | 0.8751 | 0.7536 | 0.1602 | 0.8574 | 0.7303 | 0.7138 | 0.8345 | 0.8623 | 0.9066 | 0.5296 | 0.7223 |
|  | *H*_S_ | 0.875 | 0.754 | 0.161 | 0.862 | 0.732 | 0.716 | 0.836 | 0.864 | 0.912 | 0.531 | 0.7243 |
|  | *F*_IS_ | 0.0032 | 0.0120 | 0.4713 | 0.5210 | 0.2579 | 0.3467 | 0.1941 | 0.1625 | 0.4992 | 0.1983 | 0.2666 |
|  | *r* | -0.0041 | 0.0079 | 0.1413 | 0.247 | 0.1209 | 0.1677 | 0.0942 | 0.0765 | 0.2427 | 0.0846 |  |
|  | P_HWE_ | 0.5763 | 0.0037 | 0.0101 | 0.0000 | 0.0031 | 0.0001 | 0.0000 | 0.0259 | 0.0000 | 0.0506 |  |
| Changchun | *N* | 10 | 10 | 10 | 10 | 10 | 10 | 9 | 10 | 10 | 10 |  |
|  | *A*/*A*_R_ | 8/5.220 | 7/5.315 | 3/2.395 | 8/5.872 | 7/5.198 | 7/5.272 | 6/4.836 | 11/7.272 | 8/5.993 | 3/2.894 | 6.8/5.027 |
|  | *H*_O_ | 0.8000 | 0.8000 | 0.2000 | 0.7000 | 0.8000 | 0.4000 | 0.5556 | 1.0000 | 0.5000 | 0.5000 | 0.6256 |
|  | *uH*_E_ | 0.7737 | 0.8105 | 0.3526 | 0.8632 | 0.8316 | 0.8211 | 0.7974 | 0.9263 | 0.8790 | 0.6474 | 0.7703 |
|  | *H*_S_ | 0.772 | 0.811 | 0.361 | 0.872 | 0.833 | 0.844 | 0.813 | 0.922 | 0.9 | 0.656 | 0.7784 |
|  | *F*_IS_ | -0.0360 | 0.0137 | 0.4462 | 0.1975 | 0.0400 | 0.5263 | 0.3162 | -0.0843 | 0.4444 | 0.2373 | 0.2101 |
|  | *r* | -0.0398 | -0.008 | 0.1593 | 0.0731 | -0.0032 | 0.2251 | 0.1326 | -0.0711 | 0.1948 | 0.1016 |  |
|  | P_HWE_ | 0.4802 | 0.8842 | 0.2989 | 0.5035 | 0.4799 | 0.0045 | 0.2604 | 0.7047 | 0.0093 | 0.5146 |  |
| Shenyang | *N* | 47 | 47 | 47 | 47 | 47 | 47 | 45 | 46 | 45 | 47 |  |
|  | *A*/*A*_R_ | 9/4.560 | 7/4.706 | 3/2.549 | 8/5.072 | 6/3.298 | 12/4.570 | 10/5.191 | 9/4.428 | 13/6.364 | 4/2.752 | 8.1/4.349 |
|  | *H*_O_ | 0.7872 | 0.7872 | 0.1915 | 0.6170 | 0.5532 | 0.6809 | 0.6000 | 0.6957 | 0.4000 | 0.6170 | 0.5930 |
|  | *uH*_E_ | 0.7692 | 0.7932 | 0.4299 | 0.8234 | 0.6321 | 0.7506 | 0.8112 | 0.7148 | 0.8871 | 0.5832 | 0.7195 |
|  | *H*_S_ | 0.769 | 0.793 | 0.432 | 0.826 | 0.633 | 0.751 | 0.814 | 0.715 | 0.893 | 0.583 | 0.7209 |
|  | *F*_IS_ | -0.0238 | 0.0076 | 0.5572 | 0.2527 | 0.1261 | 0.0939 | 0.2626 | 0.0270 | 0.5519 | -0.0587 | 0.1797 |
|  | *r* | -0.0125 | -0.0144 | 0.2224 | 0.1183 | 0.0572 | 0.0393 | 0.1113 | 0.0229 | 0.2671 | -0.0381 |  |
|  | P_HWE_ | 0.2609 | 0.0692 | 0.0000 | 0.0211 | 0.3361 | 0.2115 | 0.0000 | 0.4661 | 0.0000 | 0.9736 |  |
| Qingtongxia | *N* | 7 | 7 | 7 | 7 | 7 | 7 | 7 | 7 | 5 | 7 |  |
|  | *A*/*A*_R_ | 6/5.340 | 4/3.428 | 2/1.714 | 5/4.571 | 5/4.571 | 4/3.582 | 2/1.714 | 6/5.121 | 5/5.000 | 3/2.714 | 4.2/3.776 |
|  | *H*_O_ | 0.8571 | 0.5714 | 0.1429 | 0.4286 | 1.0000 | 0.5714 | 0.1429 | 0.8571 | 0.2000 | 0.2857 | 0.5057 |
|  | *uH*_E_ | 0.8571 | 0.6264 | 0.1429 | 0.7802 | 0.7802 | 0.5824 | 0.1429 | 0.8242 | 0.8667 | 0.6044 | 0.6207 |
|  | *H*_S_ | 0.857 | 0.631 | 0.143 | 0.81 | 0.762 | 0.583 | 0.143 | 0.821 | 0.95 | 0.631 | 0.6331 |
|  | *F*_IS_ | 0.0000 | 0.0943 | — | 0.4706 | -0.3125 | 0.0204 | — | -0.0435 | 0.7895 | 0.5472 | 0.1566 |
|  | *r* | -0.0475 | -0.0145 | -0.0742 | 0.1966 | -0.25 | -0.0059 | -0.0742 | -0.0869 | 0.3523 | 0.2351 |  |
|  | P_HWE_ | 0.9387 | 1.0000 | — | 0.0749 | 0.9391 | 0.6743 | — | 0.1418 | 0.0041 | 0.1168 |  |
| Qingdao | *N* | 47 | 47 | 47 | 46 | 47 | 45 | 43 | 46 | 47 | 47 |  |
|  | *A*/*A*_R_ | 8/4.187 | 7/5.130 | 2/1.818 | 13/5.837 | 7/4.822 | 10/4.439 | 13/4.183 | 14/6.298 | 11/5.632 | 4/3.144 | 8.9/4.549 |
|  | *H*_O_ | 0.7447 | 0.7234 | 0.1702 | 0.4565 | 0.8298 | 0.6444 | 0.4884 | 0.8261 | 0.5957 | 0.6170 | 0.6096 |
|  | *uH*_E_ | 0.6985 | 0.8309 | 0.2562 | 0.8557 | 0.8035 | 0.6979 | 0.5951 | 0.8863 | 0.8367 | 0.6308 | 0.7091 |
|  | *H*_S_ | 0.698 | 0.832 | 0.257 | 0.86 | 0.803 | 0.698 | 0.596 | 0.887 | 0.839 | 0.631 | 0.7101 |
|  | *F*_IS_ | -0.0669 | 0.1306 | 0.3381 | 0.4693 | -0.0331 | 0.0774 | 0.1811 | 0.0686 | 0.2902 | 0.0220 | 0.1477 |
|  | *r* | -0.0596 | 0.0595 | 0.1278 | 0.2297 | -0.0251 | 0.0165 | 0.1006 | 0.0299 | 0.1416 | 0.0114 |  |
|  | P_HWE_ | 0.6502 | 0.5945 | 0.0461 | 0.0000 | 0.4497 | 0.2989 | 0.0001 | 0.4760 | 0.0018 | 0.4018 |  |
| Taian | *N* | 41 | 41 | 41 | 41 | 41 | 40 | 41 | 39 | 39 | 41 |  |
|  | *A*/*A*_R_ | 10/4.586 | 7/4.941 | 4/2.665 | 13/5.519 | 9/5.502 | 11/5.263 | 11/3.073 | 10/5.037 | 12/6.402 | 3/2.537 | 9.0/4.553 |
|  | *H*_O_ | 0.6585 | 0.7317 | 0.2195 | 0.3171 | 0.8293 | 0.8000 | 0.4390 | 0.6410 | 0.5641 | 0.4146 | 0.5615 |
|  | *uH*_E_ | 0.7133 | 0.8094 | 0.4219 | 0.8299 | 0.8422 | 0.8193 | 0.4059 | 0.8042 | 0.8898 | 0.5288 | 0.7065 |
|  | *H*_S_ | 0.714 | 0.81 | 0.424 | 0.836 | 0.842 | 0.82 | 0.405 | 0.806 | 0.894 | 0.53 | 0.7081 |
|  | *F*_IS_ | 0.0777 | 0.0971 | 0.4828 | 0.6209 | 0.0156 | 0.0239 | -0.0827 | 0.2050 | 0.3691 | 0.2179 | 0.2027 |
|  | *r* | 0.0215 | 0.0447 | 0.1924 | 0.2999 | -0.0024 | -0.0029 | -0.0802 | 0.0984 | 0.1743 | 0.0982 |  |
|  | P_HWE_ | 0.5990 | 0.1509 | 0.0003 | 0.0000 | 0.6854 | 0.1553 | 0.8601 | 0.1022 | 0.0000 | 0.1775 |  |
| Baoshan | *N* | 48 | 48 | 48 | 46 | 48 | 48 | 47 | 48 | 48 | 48 |  |
|  | *A*/*A*_R_ | 11/5.290 | 7/4.785 | 4/1.989 | 12/5.594 | 9/4.447 | 11/4.731 | 10/5.007 | 12/6.026 | 16/6.339 | 5/2.952 | 9.7/4.716 |
|  | *H*_O_ | 0.8333 | 0.7708 | 0.2083 | 0.3044 | 0.8333 | 0.6458 | 0.5319 | 0.8958 | 0.6458 | 0.6042 | 0.6274 |
|  | *uH*_E_ | 0.8274 | 0.7954 | 0.2436 | 0.8199 | 0.7634 | 0.7693 | 0.8092 | 0.8708 | 0.8669 | 0.6042 | 0.7370 |
|  | *H*_S_ | 0.827 | 0.796 | 0.244 | 0.826 | 0.763 | 0.771 | 0.812 | 0.871 | 0.869 | 0.604 | 0.7383 |
|  | *F*_IS_ | -0.0072 | 0.0312 | 0.1462 | 0.6314 | -0.0927 | 0.1619 | 0.3451 | -0.0290 | 0.2570 | -0.0000 | 0.1444 |
|  | *r* | -0.0101 | 0.0136 | 0.074 | 0.3044 | -0.0558 | 0.0649 | 0.1612 | -0.0218 | 0.1279 | -0.0114 |  |
|  | P_HWE_ | 0.2974 | 0.6954 | 0.1027 | 0.0000 | 0.4226 | 0.0000 | 0.0000 | 0.7603 | 0.0000 | 0.4572 |  |
| Dali | *N* | 30 | 30 | 30 | 30 | 30 | 29 | 30 | 29 | 28 | 30 |  |
|  | *A*/*A*_R_ | 11/5.130 | 7/4.890 | 3/2.049 | 13/6.736 | 9/5.143 | 12/5.754 | 11/5.793 | 15/6.760 | 15/6.616 | 4/3.034 | 10/5.191 |
|  | *H*_O_ | 0.7333 | 0.7333 | 0.2333 | 0.4000 | 0.7667 | 0.6552 | 0.5333 | 0.8276 | 0.5357 | 0.5333 | 0.5952 |
|  | *uH*_E_ | 0.8006 | 0.7904 | 0.2672 | 0.9045 | 0.8243 | 0.8294 | 0.8356 | 0.9020 | 0.8721 | 0.6107 | 0.7637 |
|  | *H*_S_ | 0.802 | 0.791 | 0.268 | 0.913 | 0.825 | 0.833 | 0.841 | 0.903 | 0.878 | 0.612 | 0.7666 |
|  | *F*_IS_ | 0.0853 | 0.0733 | 0.1288 | 0.5620 | 0.0710 | 0.2130 | 0.3657 | 0.0838 | 0.3901 | 0.1286 | 0.2102 |
|  | *r* | 0.0392 | 0.0206 | 0.0431 | 0.2715 | 0.0251 | 0.1015 | 0.1729 | 0.0354 | 0.1766 | 0.038 |  |
|  | P_HWE_ | 0.7284 | 0.2087 | 0.4988 | 0.0000 | 0.5467 | 0.0080 | 0.0000 | 0.1343 | 0.0000 | 0.1657 |  |
| Kunming | *N* | 48 | 48 | 48 | 48 | 48 | 48 | 47 | 47 | 48 | 47 |  |
|  | *A*/*A*_R_ | 13/5.879 | 7/4.686 | 3/2.569 | 12/5.880 | 11/4.857 | 10/5.237 | 13/5.439 | 13/5.154 | 16/6.757 | 4/2.870 | 10.2/4.933 |
|  | *H*_O_ | 0.8125 | 0.7500 | 0.2292 | 0.6042 | 0.8542 | 0.7500 | 0.5106 | 0.7021 | 0.4583 | 0.4681 | 0.6139 |
|  | *uH*_E_ | 0.8570 | 0.7781 | 0.4362 | 0.8557 | 0.7561 | 0.8202 | 0.8145 | 0.8122 | 0.8989 | 0.5603 | 0.7589 |
|  | *H*_S_ | 0.857 | 0.778 | 0.438 | 0.858 | 0.755 | 0.821 | 0.818 | 0.813 | 0.904 | 0.561 | 0.7603 |
|  | *F*_IS_ | 0.0525 | 0.0364 | 0.4772 | 0.2962 | -0.1312 | 0.0864 | 0.3756 | 0.1368 | 0.4928 | 0.1660 | 0.1989 |
|  | *r* | 0.0212 | 0.0117 | 0.1994 | 0.1406 | -0.0874 | 0.0368 | 0.1866 | 0.0577 | 0.2385 | 0.08 |  |
|  | P_HWE_ | 0.7294 | 0.7539 | 0.0001 | 0.0086 | 0.6218 | 0.0289 | 0.0000 | 0.0402 | 0.0000 | 0.0785 |  |

*N*, number of samples; *A*, number of alleles; *A*_R_, allelic richness corrected for 5 diploid individuals; *H*_O_, observed heterozygosity; *uH*_E_, unbiased expected heterozygosity, *H*_S_, gene diversity, *F*_IS_, inbreeding coefficient, *r*, frequency of null alleles.
