# Supplementary material for: Invasion Genetics of the Western Flower Thrips in China: Evidence for Genetic Bottleneck, Hybridization and Bridgehead Effect
Source: PLoS One. 2012 Apr 3;7(4):e34567. doi: 10.1371/journal.pone.0034567 (PMC3317996; doi:10.1371/journal.pone.0034567)
Supplement: Table S2 — Basic indices calculated using COI gene and ten microsatellites based only on WFTG individuals and haplotype distribution in Chinese populations. (DOC) [file pone.0034567.s002.doc]

**Table S2.** Basic indices calculated using COI gene and ten microsatellites based only on WFTG individuals and haplotype distribution in Chinese populations.

| POP | mtDNA | | | | | | | | microsatellites | | | | |
| --- | --- | --- | --- | --- | --- | --- | --- | --- | --- | --- | --- | --- | --- |
|  | *N*_h_ | Hap1 | Hap2 | Hap3 | Hap5 | *H*_d_ (±SD) | k | π (±SD) | *A*_P_ | *A*_R_ | *H*_O_ | *uH*_E_ | *H*_S_ |
| BJ | 3 | 29 | 15 | 4 | 0 | 0.542 (0.052) | 0.595 | 0.00104 (0.00013) | 0 | 4.517 | 0.531 | 0.727 | 0.729 |
| DH | 3 | 14 | 0 | 8 | 0 | 0.565 (0.071) | 3.493 | 0.00612 (0.00312) | 2 | 4.512 | 0.57 | 0.718 | 0.721 |
| GY | 3 | 17 | 1 | 12 | 0 | 0.536 (0.048) | 0.563 | 0.00099 (0.00012) | 0 | 4.361 | 0.586 | 0.689 | 0.691 |
| JQ | 2 | 30 | 0 | 5 | 0 | 0.252 (0.085) | 0.252 | 0.00044 (0.00015) | 5 | 4.728 | 0.654 | 0.75 | 0.751 |
| HRB | 3 | 40 | 1 | 3 | 0 | 0.172 (0.074) | 0.175 | 0.00031 (0.00013) | 2 | 4.707 | 0.613 | 0.733 | 0.734 |
| QHD | 3 | 40 | 3 | 4 | 0 | 0.270 (0.081) | 0.281 | 0.00049 (0.00015) | 1 | 4.817 | 0.541 | 0.722 | 0.724 |
| CC | 2 | 8 | 0 | 0 | 0 | 0.356 (0.159) | 6.756 | 0.01183 (0.00529) | 0 | 5.027 | 0.626 | 0.77 | 0.778 |
| SY | 4 | 29 | 13 | 4 | 0 | 0.547 (0.060) | 1.377 | 0.00241 (0.00133) | 1 | 4.349 | 0.593 | 0.72 | 0.721 |
| QTX | 2 | 4 | 0 | 0 | 0 | 0.571 (0.119) | 10.857 | 0.01901 (0.00398) | 0 | 3.776* | 0.506 | 0.621 | 0.633 |
| QD | 4 | 27 | 9 | 10 | 0 | 0.600 (0.057) | 1.467 | 0.00257 (0.00133) | 1 | 4.549 | 0.61 | 0.709 | 0.71 |
| TA | 3 | 19 | 12 | 10 | 0 | 0.656 (0.035) | 0.802 | 0.00141 (0.00013) | 1 | 4.553 | 0.562 | 0.707 | 0.708 |
| BS | 4 | 19 | 21 | 6 | 0 | 0.648 (0.037) | 2.276 | 0.00399 (0.00175) | 1 | 4.716 | 0.627 | 0.737 | 0.738 |
| DL | 4 | 13 | 3 | 13 | 1 | 0.634 (0.049) | 0.761 | 0.00133 (0.00018) | 0 | 5.191 | 0.595 | 0.764 | 0.767 |
| KM | 4 | 27 | 7 | 13 | 0 | 0.601 (0.053) | 1.449 | 0.00254 (0.00131) | 2 | 4.933 | 0.614 | 0.759 | 0.76 |
| Total |  | 316 | 85 | 92 | 1 |  |  |  |  |  |  |  |  |
| Mean |  |  |  |  |  | 0.496 |  |  |  | 4.624 | 0.588 | 0.723 | 0.726 |

*N*_h_, number of haplotypes; *H*_d_, haplotype diversity; k, average number of nucleotide differences; π, nucleotide diversity; *A*_P_, number of private alleles; *A*_R_, allelic richness; *H*_O_, observed heterozygosity; *uH*_E_, unbiased expected heterozygosity; *H*_S_, gene diversity; *Allelic richness estimates presented without rarefaction because population had fewer than 5 individuals.
