# Supplementary material for: Invasion Genetics of the Western Flower Thrips in China: Evidence for Genetic Bottleneck, Hybridization and Bridgehead Effect
Source: PLoS One. 2012 Apr 3;7(4):e34567. doi: 10.1371/journal.pone.0034567 (PMC3317996; doi:10.1371/journal.pone.0034567)
Supplement: Table S3 — Pairwise FST matrix obtained using 10 microsatellite loci. All WFTL individuals were omitted from the respective population and were treated as a single population. (DOC) [file pone.0034567.s003.doc]

**Table S3.** Pairwise *F_ST_* matrix obtained using 10 microsatellite loci. All WFTL individuals were omitted from the respective population and were treated as a single population.

|  | BJ | DH | GY | JQ | HRB | QHD | CC | SY | QTX | QD | TA | BS | DL | KM |
| --- | --- | --- | --- | --- | --- | --- | --- | --- | --- | --- | --- | --- | --- | --- |
| DH | **0.039** |  |  |  |  |  |  |  |  |  |  |  |  |  |
| GY | **0.057** | **0.060** |  |  |  |  |  |  |  |  |  |  |  |  |
| JQ | **0.072** | **0.083** | **0.064** |  |  |  |  |  |  |  |  |  |  |  |
| HRB | **0.025** | **0.037** | **0.043** | **0.058** |  |  |  |  |  |  |  |  |  |  |
| QHD | **0.033** | **0.040** | **0.039** | **0.063** | **0.020** |  |  |  |  |  |  |  |  |  |
| CC | 0.018 | 0.004 | 0.024 | **0.045** | 0.010 | 0.014 |  |  |  |  |  |  |  |  |
| SY | **0.066** | **0.057** | **0.062** | **0.082** | **0.052** | **0.030** | 0.033 |  |  |  |  |  |  |  |
| QTX | 0.052 | 0.093 | 0.080 | **0.088** | 0.051 | 0.045 | 0.048 | **0.085** |  |  |  |  |  |  |
| QD | **0.067** | **0.050** | **0.060** | **0.097** | **0.062** | **0.055** | 0.003 | **0.056** | **0.083** |  |  |  |  |  |
| TA | **0.053** | **0.056** | **0.058** | **0.079** | **0.049** | **0.052** | 0.015 | **0.068** | 0.075 | **0.038** |  |  |  |  |
| BS | **0.022** | 0.019 | **0.028** | **0.062** | **0.018** | **0.015** | -0.008 | **0.033** | 0.064 | **0.033** | **0.041** |  |  |  |
| DL | **0.027** | **0.033** | **0.023** | **0.060** | **0.028** | 0.010 | -0.001 | **0.034** | 0.047 | **0.029** | **0.033** | 0.008 |  |  |
| KM | **0.025** | 0.017 | **0.037** | **0.058** | **0.014** | **0.018** | -0.004 | **0.040** | 0.053 | **0.045** | **0.032** | 0.012 | 0.012 |  |
| WFTL | 0.021 | 0.018 | 0.021 | **0.069** | 0.017 | 0.012 | -0.002 | 0.027 | 0.048 | 0.022 | 0.030 | 0.000 | 0.001 | 0.008 |

Bold indicates significant indices after Bonferroni correction (P = 0.05).
