# Supplementary material for: Invasion Genetics of the Western Flower Thrips in China: Evidence for Genetic Bottleneck, Hybridization and Bridgehead Effect
Source: PLoS One. 2012 Apr 3;7(4):e34567. doi: 10.1371/journal.pone.0034567 (PMC3317996; doi:10.1371/journal.pone.0034567)
Supplement: Table S4 — Results of assignment test and detection of first generation migrants (F0) based on WFTG individuals, with source populations list by column and recipient populations by row. Populations with sample size of ≤10 individuals were not included. (DOC) [file pone.0034567.s004.doc]

**Table S4.** Results of assignment test and detection of first generation migrants (F0) based on WFTG individuals, with source populations list by column and recipient populations by row. Populations with sample size of ≤ 10 individuals were not included.

|  | BJ | DH | GY | JQ | HRB | QHD | SY | QD | TA | BS | DL | KM |
| --- | --- | --- | --- | --- | --- | --- | --- | --- | --- | --- | --- | --- |
| BJ | **30** | 1 |  |  |  | 1 |  |  | 1 | 1 | 7(1) | 7(1) |
| DH |  | **18** |  |  |  |  |  |  |  | (1) | 2 | 2 |
| GY |  |  | **20** |  |  |  |  | 1 |  |  | 9(1) |  |
| JQ |  |  |  | **34** |  |  |  |  |  |  | 1(1) |  |
| HRB | 1 |  |  |  | **29** | 3(1) |  | 1(1) |  |  | 6 | 4 |
| QHD |  | 1 |  |  |  | **29** | 1 |  |  | 2 | 10(3) | 4(1) |
| SY |  |  |  |  | 1 | 2 | **28** | (1) |  | 1 | 12(1) | 2 |
| QD |  |  |  |  |  | (1) | 1 | **37** |  | 1 | 5(1) | 2 |
| TA |  | 1 |  |  |  |  |  |  | **28** |  | 8(1) | 4 |
| BS |  |  |  |  |  | 3 |  |  |  | **29** | 12(2) | 2 |
| DL |  |  |  |  |  |  |  |  | 1 | (3) | **28** | 1 |
| KM |  | 1 |  |  |  | (1) | 1 |  |  | 1(2) | 5(1) | **39** |
