# Supplementary material for: Invasion Genetics of the Western Flower Thrips in China: Evidence for Genetic Bottleneck, Hybridization and Bridgehead Effect
Source: PLoS One. 2012 Apr 3;7(4):e34567. doi: 10.1371/journal.pone.0034567 (PMC3317996; doi:10.1371/journal.pone.0034567)
Supplement: Table S5 — Within-population tests for heterozygosity excess P-values. (DOC) [file pone.0034567.s005.doc]

**Table S5.** Within-population tests for Heterozygosity excess P-values.

| **Populations** | **Heterozygosity excess P-values** | | |
| --- | --- | --- | --- |
|  | **IAM** | **TPM** | **SMM** |
| Beijing | **0.00342** | 0.21582 | 0.98389 |
| Dunhuang | 0.08008 | 0.57715 | 0.95801 |
| Guiyang | 0.09668 | 0.57715 | 0.98389 |
| Jiuquan | **0.00049** | 0.21582 | 0.99512 |
| Harbin | **0.00684** | 0.50000 | 0.99658 |
| Qinghuangdao | 0.05273 | 0.57715 | 0.99951 |
| Changchun | 0.13770 | 0.24609 | 0.83887 |
| Shenyang | **0.00244** | 0.27832 | 0.95801 |
| Qingtongxia | 0.27832 | 0.61523 | 0.75391 |
| Qingdao | 0.08008 | 0.34766 | 0.91992 |
| Taian | 0.09668 | 0.57715 | 0.98389 |
| Baoshan | **0.01221** | 0.72168 | 0.99902 |
| Dali | **0.00342** | 0.68750 | 0.99316 |
| Kunming | **0.00098** | 0.50000 | 0.99902 |

Bold indicates significant at P = 0.05
