# Supplementary material for: Invasion Genetics of the Western Flower Thrips in China: Evidence for Genetic Bottleneck, Hybridization and Bridgehead Effect
Source: PLoS One. 2012 Apr 3;7(4):e34567. doi: 10.1371/journal.pone.0034567 (PMC3317996; doi:10.1371/journal.pone.0034567)
Supplement: Table S6 — Results of AMOVA test on mitochondrial and microsatellite markers. (DOC) [file pone.0034567.s006.doc]

**Table S6.** Results of AMOVA test on mitochondrial and microsatellite markers

| Groups | Source of variation | mtDNA | | | Microsatellites | | |
| --- | --- | --- | --- | --- | --- | --- | --- |
|  |  | Variance components | % variation | Fixation indicies | Variance components | % variation | Fixation indicies |
| Two groups (3 southwestern pops/ rest pops of China) | Among groups | -0.0149 Va | -2.04 | FCT = -0.0204 (P =0.4787) | -0.02780 Va | -0.75 | FCT = -0.0075(P =0.9643) |
|  | Among pops within groups | 0.0949 Vb | 13 | FSC = 0.1274 (P < 0.0001) | 0.16758 Vb | 4.53 | FSC = 0.0450(P < 0.0001) |
|  | Within populations | 0.6501 Vc | 89.04 | FST = 0.1096 (P < 0.0001) | 3.55863 Vc | 96.22 | FST = 0.0378(P < 0.0001) |
| Two groups (Two STRUCTURE clusters) | Among groups | -0.0131 Va | -1.79 | FCT = -0.0179 (P =0.5912) | 0.02244 Va | 0.6 | FCT = 0.0060(P =0.0060) |
|  | Among pops within groups | 0.0957 Vb | 13.06 | FSC = 0.1283 (P < 0.0001) | 0.14461 Vb | 3.88 | FSC = 0.0391(P < 0.0001) |
|  | Within populations | 0.6501 Vc | 88.73 | FST = 0.1127 (P < 0.0001) | 3.55863 Vc | 95.52 | FST = 0.0448(P < 0.0001) |
| Two groups (WFTG/ WFTL) | Among groups |  |  |  | -0.08508 Va | -2.2882 | FCT = -0.0229(P =0.9970) |
|  | Among pops within groups |  |  |  | 0.16197 Vb | 4.3559 | FSC = 0.0426(P < 0.0001) |
|  | Within populations |  |  |  | 3.64146 Vc | 97.9323 | FST = 0.0207(P < 0.0001) |
